# Notices

**Published:** 1868-03

**Authors:** 


The remainder of the preceding article on Bronchitis and Kindred Diseases with cases and illustrations, will appear in the April number. Each number of the Journal contains on an average twenty pages of regular reading matter of general interest; mere advertisements will not be allowed to intrench on this space; last year, over 260 pages were given.

---

*NOTICES.*——" London Lancet," reprinted monthly by Wm. C. Herald, No. 32 Beekman St. New York, $5 a year. The trade is supplied by the American News Co., 121 Nassau St. New York. The January No. contains a lecture on Woman's Diseases by J. Braxton Hicks, M. D. F. R. S. The Anatomy and Surgery of the Human Foot, by Henry Hancock, F. R. C. S. Lateral Curvature of the Spine, by Richard Barwell, F. R. C. S. Propagation of Consumption, by Wm. Budd, M. D. Stone in the Bladder, by Sir Henry Thompson. Diptheria treated by Iodine Inhalation, by J. Warren Curren. Jefferson's New Apparatus for Fractures. Nicotine for Traumatic Tetanus, which being interpreted means Lock Jaw treated by Tobacco. Lime for Cancerous and other internal tumors. Ashton on Practical Surgery. Carbolic Acid for Burns, with Hospital Reports, Medical Annotations, Reviews, Notices, &c. of Medical Books; a very valuable number.

Scribner, Wellford & Co. and Warne & Co. London, have published "Domestic Medicine and Surgery," with a glossary of the terms used therein by J. H. Walsh, F.R.C.S., author of a Manual of Domestic Economy, the Fourth Thousand, illustrated with numerous wood engravings and colored plates, 722 p.p. 12 mo. The value of the book for popular instruction may be learned from the table of the opening chapters: 1st, General Principles of maintaining health and removing disease; 2d, Maintenance of Health by means external to the body; 3d, by internal means. These three chapters embrace a large amount of valuable information about diet, drinks; solid and liquid food; mental occupation; exercise of body and mind; cleanliness; lighting; ventilation; weather; warmth; climate. habits, &c., &c.; a valuable book not only for families but for young physicians; and the old as well, as it brings them up to the knowledge of the present times, its discoveries, improvements and progress. $5.25 by mail.

MODEL COOKERY.—A still more valuable publication by the same House is "Warner's Model Cookery" and House-keeping Book, containing complete instruction in household management; with three thousand receipts pertaining to food, household matters, servants, &c., compiled and edited by Mary Jewry, with original illustrations printed in colors by Kronbeim, 728 pp. 12mo., with a full analytical index of pages of three columns each; it is the fullest, most reliable and complete cook book ever offered to the American people, and ought to be considered an indispensable article of house-keeping by every just married couple. Address Scribner, Wellford & Co., New York. $3.75 by mail, p. p.

THE GUARDIAN OF HEALTH, edited by W. M. Cornell, M. D. L.L.D., No. 4 Hayward Place, Boston, Mass., $1 a year, single numbers 12 cents, issued monthly. No. 2, Vol. 7, for 1868, February; is a continuation of a valuable article entitled "One Primary cause of Epilepsy only." Hysterics; physical training of girls; rules for home education; necessary rules for sleep; how to prevent cold feet; bathing; death indoors; Hello! here we are, just on the point of commending the practical character and good sense of the articles which fill up this February number, when upon closer inspection, we find that some of them are our own waifs, credited to various sources. Within a few days we have seen two fugitives in different papers which we wrote while studying medicine, so long ago! that we do not care to state the year of their birth: but the "Guardian" has thirty-eight different articles, all short and all good; that one about the healthful wriggle of the pretty girls of Philadelphia for example and the Frog Story. But, Doctor, instead of scissoring around, why don't you fill every "Guardian" with original articles; for you must know "a heap" by this time; you had been making a noise in the world before we knew our A. B. C's, and putting one thing and another together, you must now be about a hundred and three years old, perhaps four; but to be on the safe side, and have a margin say, a hundred and two; during that time you must have seen sights, a good many of them; wont you begin in the next number and give us a few?

